# 

**Published:** 1862-03

**Authors:** 


### CONTENTS.---MARCH, 1862.

### ORIGINAL CONTRIBUTIONS.

PAGE

ART. VIII. OVARIAN TUMORS. By WM. H. BYFORD, A. M., M. D., Professor of Obstetrics and Diseases of Women and Children, in the Medical Department, Lind University, Chicago, Ill.,

ART. IX. Eligible Pharmaceutical forms for the Administration of Medicines. An Inaugural Thesis, by JNO. MAYNARD WOODWORTH, M. D. .......... 145

### THE CLINIQUE.

Mercy Hospital, Service Prof .E. ANDREWS, Surgeon-in-Charge. Reproduction of the Inferior Maxillary—Preservation of the Teeth.... .......... 153

### SELECTIONS.

Pyrophosphate of Iron. CHAPMAN,.......... 157

Reflex Paraplegia—Reprinted from *Braithwaite*, .......... 167

### BOOK NOTICES.

BORDER LINES OF KNOWLEDGE, in some Provinces of Medical Science. By OLIVER WENDELL HOLMES, M. D., etc.,.......... 174

COMMENTARIES on the Surgery of the War in Portugal, Spain, France and the Netherlands. By G. J. GUTHRIE, F. R. S.,.......... 179

### EDITORIAL.

American Medical Association,.......... 187

Close of the Medical College Terms in this Ciiy—Prosperity of the Colleges—Prospects for the Future,.......... 188

Death of Dr. J. N. GRAHAM., .......... 189

SPECIAL SELECTIONS:—The Country Practitioner,.......... 190

American Journal of Ophthalmology, .......... 191

Temperature and Ventilation,.......... 191

Food in Typhoid Fever,.......... 192

Sufferings of the Wounded at Fort Donelson,.......... 192

